# Ecosystem Services Transcend Boundaries: Estuaries Provide Resource Subsidies and Influence Functional Diversity in Coastal Benthic Communities

**DOI:** 10.1371/journal.pone.0042708

**Published:** 2012-08-03

**Authors:** Candida Savage, Simon F. Thrush, Andrew M. Lohrer, Judi E. Hewitt

**Affiliations:** 1 Department of Marine Science, University of Otago, Dunedin, New Zealand; 2 National Institute of Water and Atmospheric Research, Hamilton, New Zealand; University of Connecticut, United States of America

## Abstract

**Background:**

Estuaries are highly productive ecosystems that can export organic matter to coastal seas (the ‘outwelling hypothesis’). However the role of this food resource subsidy on coastal ecosystem functioning has not been examined.

**Methodology/Principal Findings:**

We investigated the influence of estuarine primary production as a resource subsidy and the influence of estuaries on biodiversity and ecosystem functioning in coastal mollusk-dominated sediment communities. Stable isotope values (δ^13^C, δ^15^N) demonstrated that estuarine primary production was exported to the adjacent coast and contributed to secondary production up to 4 km from the estuary mouth. Further, isotope signatures of suspension feeding bivalves on the adjacent coast (*Dosinia subrosea*) closely mirrored the isotope values of the dominant bivalves inside the estuaries (*Austrovenus stutchburyi*), indicating utilization of similar organic matter sources. However, the food subsidies varied between estuaries; with estuarine suspended particulate organic matter (SPOM) dominant at Tairua estuary, while seagrass and fringing vegetation detritus was proportionately more important at Whangapoua estuary, with lesser contributions of estuarine SPOM. Distance from the estuary mouth and the size and density of large bivalves (*Dosinia* spp.) had a significant influence on the composition of biological traits in the coastal macrobenthic communities, signaling the potential influence of these spatial subsidies on ecosystem functioning.

**Conclusions/Significance:**

Our study demonstrated that the locations where ecosystem services like productivity are generated are not necessarily where the services are utilized. Further, we identified indirect positive effects of the nutrient subsidies on biodiversity (the estuarine subsidies influenced the bivalves, which in turn affected the diversity and functional trait composition of the coastal sediment macrofaunal communities). These findings highlight the importance of integrative ecosystem-based management that maintains the connectivity of estuarine and coastal ecosystems.

## Introduction

Trophic subsidies between terrestrial and coastal marine ecosystems play a key role in connectivity of populations and can strongly influence community dynamics in adjacent ecosystems [1], [2]. Nutrient subsidies usually increase primary and secondary productivity in the recipient habitat, both directly and indirectly [2]. Estuaries are highly productive ecosystems [3] with primary production contributed by phytoplankton, benthic microphytes, emergent and fringing vegetation, macroalgae as well as inputs of organic matter from rivers. This high production is one of the reasons that numerous fish and invertebrate species use estuaries as nursery habitats [4]. In estuaries where a high proportion of the water volume is exchanged with the tide, organic matter is exported to coastal seas. This ‘outwelling hypothesis’ [5] predicts the transfer of organic matter and nutrients across ecosystem boundaries and their incorporation by organisms in the receiving ecosystem. However, the role of estuarine primary production in supporting suspension feeding bivalves in the adjacent open coast and the influence of this trophic subsidy on functional diversity of coastal benthic communities remain poorly understood.

Long-lived suspension feeding bivalves are often important food resources in marine food webs and play important roles in biogeochemical processes and ecosystem functioning [6], [7]. However, there has been substantial loss of shellfish in estuaries and coastal ecosystems due to human activity [8], [9], prompting conservation and management efforts to maintain and restore these important ecosystem services.

The condition and growth of bivalves is dependent upon the quantity and quality of the food resources they assimilate over time. Stable isotopes (δ^13^C, δ^15^N) can provide time-integrated information about the source of food assimilated by mollusks [10], [11], [12]. If estuarine and open coast primary food resources have distinct isotopic signatures (e.g. [13]), the spatial subsidy of estuarine primary production and its contribution to secondary production can be assessed using stable isotopes. Further, isotope ratios in combination with mixing models [14] can be used to assess the relative importance of different organic matter sources that contribute to an animal's diet.

This study investigated whether the ecosystem services generated in estuaries provide food subsidies and benefit communities in adjacent coastal ecosystems. Accordingly, our overall objective was to quantify the importance of estuarine primary production to mollusk-dominated open coast sediments and determine how estuarine subsidies influence the functional diversity of the coastal communities. We used a gradient design [15], with distance from the estuary mouth as a proxy for the degree of estuarine influence. We hypothesized that the contribution of estuarine food resources in bivalve tissues would decrease with distance from the mouth of the estuary. Further, we predicted that the functional diversity of the community (as indicated by biological traits analysis) would change significantly along this estuary-to-coast gradient. To test these hypotheses, we sampled carbonate sediment communities, characterized their functional traits, and analyzed stable isotope ratios of primary producers and foot muscle tissues of the dominant coastal bivalve (*Dosinia subrosea*) across estuary-coast gradients. Foot muscle of *D. subrosea* provides information on the annual averaged diet (authors unpublished). Finally, we compared the isotope signatures of the open coast bivalves to those of the dominant bivalve inside the estuaries, *Austrovenus stutchburyi*, to assess whether these two venerid bivalve species use similar organic matter sources.

## Methods

### Study sites and sampling

Two locations <50 km apart on the east coast of the Coromandel Peninsula, North Island, New Zealand, were selected for study as they have estuaries that discharge onto a shallow sandy shelf system dominated by suspension feeding bivalves (*Dosinia* spp.). The two estuaries, Whangapoua (−36.7151: 175.6373) and Tairua (−37.0172: 175.8856), are both barrier-enclosed lagoons with extensive intertidal flats (>50% intertidal area) and an average depth of only 1.3 m. These physiographic features result in both estuaries having short residence times and non-stratified waters. Whangapoua estuary contains significant areas of mangrove (23%), seagrass (23%), saltmarsh (10%), mud- and sandflats (30%). Tairua estuary contains seagrass (20%), saltmarsh (9%), mangrove (5%), and mud- and sandflats (46%). Whangapoua estuary is about twice the area (13.1 km^2^) of Tairua estuary (6.1 km^2^), while Tairua has a larger catchment (281.7 km^2^) than Whangapoua (107.1 km^2^). Land-use in the Whangapoua catchment is dominated by pine plantation forestry (38%), indigenous forest (20%) with the rest predominantly pasture and scrubland. Indigenous forest (54%) dominates the catchment at Tairua, with shrubland, pasture and plantation forest making up the remainder of the land-use. Mean annual river discharge at Tairua is 21.2 m^3^ s^−1^ compared to 6.1 m^3^ s^−1^ at Whangapoua, while the spring tidal prism is 14.9×10^6^ m^3^ at Whangapoua and 7.7×10^6^ m^3^ at Tairua [16], [17]. Tairua offshore sites have more wave energy than Whangapoua offshore sites due to the orientation of the coast with Tairua more exposed to open ocean swells (authors personal observation).

Sampling was conducted between March and May 2010. On the open coast adjacent to both Whangapoua and Tairua estuaries, nine subtidal sites (10–15 m deep) were positioned approximately 200–300 m apart at increasing distances from the estuary mouth, with site 1 located nearest the estuary mouth (Fig. S1). At each of the 9 sites per location, 15 sediment cores (10 cm internal diameter, 13 cm deep) and 10 sediment quadrats (50×50 cm, 15 cm deep) were collected at regular intervals along a 20 m transect. The sediment cores were sieved on a 500 µm mesh and used to characterize macrofaunal diversity. The quadrats were collected using a suction dredge with an attached 5 mm mesh bag to sample the large bivalves that are often under-represented in small cores. Core and quadrat samples were preserved in 70% isopropyl alcohol and stained with Rose Bengal. All *Dosinia* spp. (the dominant surf clam) present in the dredge samples were measured to the nearest mm using digital calipers to determine population size structure. Three to five individuals of *Dosinia subrosea* per site were retained when available and stored frozen until analysis for isotopic signatures (see below). All necessary permits were obtained for the described field studies.

To characterize the sediment grain size, organic and carbonate content, and chlorophyll *a* (chl *a*) and phaeophytin concentrations, triplicate cores (1.5 cm diameter×2 cm depth) were collected from each end of the 20 m transect samples at each subtidal site. The six cores collected at each site were kept chilled and in the dark until they were frozen (−20°C). Prior to analysis, samples were thawed, pooled and homogenized to provide one representative value for each environmental factor per site. Grain size samples were digested in 9% hydrogen peroxide for 48 hours to remove organic matter. Wet sieving was used to measure cumulative % weights of gravel, coarse sand, medium sand, fine sand, sediment fractions (i.e., particles sizes >2; 2 – 0.5; 0.5 – 0.25; 0.25-0.063 mm diameter respectively) and pipette analysis was used to measure the silt and clay fraction (0.063 - 0.0039 and <0.0039 mm diameter, respectively). Chl *a* was extracted from freeze-dried sediments by boiling in 90% ethanol and measured spectrophotometrically. An acidification step was included to separate degradation products (phaeophytin). Organic matter content was determined as loss-on-ignition at 400°C for 5.5 hours. Sediment carbonate concentrations were measured using gasometric quantitative analysis, with a precision of ±2% [18]. This method measures the carbonate concentration of the sand or finer particle sizes, neither site exhibited a large proportion of coarse shell hash.

Potential estuarine food resources were collected within Whangapoua and Tairua estuaries to characterize the isotopic signature of estuarine primary producers. To account for spatial variability within each estuary, replicate samples (n = 3–5) of primary producers were collected at three to five sites distributed across the estuary using a transect sampling approach [19]. The collected materials included phytoplankton (suspended particulate organic matter, SPOM), microphytobenthos (MPB), seagrass (*Zostera capricorni*), mangrove (*Avicennia marina*) leaves, and saltmarsh plants. Three liters of water, containing phytoplankton, were collected from a tidal channel in the center of each estuary and from the adjacent coast (water depth >10 m) to test for differences in SPOM isotopic signatures between estuarine and marine end-members. The water samples were filtered (Whatmann GF/F 0.45 µm) and isotopic signatures determined for the SPOM retained on the filters. MPB was sampled using opaque sediment cores (5 cm internal diameter, three cores per site) that were kept upright and in the dark for 6–8 hours. A double layer of filter paper was placed on the sediment surface inside each core and the cores were exposed to overhead light for another 10 hours, stimulating the MPB to migrate upwards into the filter paper. The filter paper containing MPB was frozen at −20°C and kept in darkness until analysis. Vegetation samples (seagrass, mangrove, saltmarsh) were placed in separate plastic bags, cleaned of epiphytes, and stored frozen until analysis. The isotopic composition of estuarine food resources from each site were analyzed separately but are pooled here to obtain an integrated isotopic signature of primary producers across the estuary for each study area. In addition, a sample of drifting seagrass detritus from a subtidal coastal site (>10 m) off Whangapoua was obtained for isotope analysis. The mean isotope values of this sample (analyzed in triplicate) and fresh seagrass from within Whangapoua estuary were used in the mixing models to assess the contribution of seagrass detritus to offshore bivalves at Whangapoua. At Tairua, fresh seagrass detritus collected within Tairua estuary was used in the mixing model with a correction for fractionation associated with degradation (see below). To provide an integrated value of the estuarine food resources potentially available to open coast bivalves we also sampled individuals of the venerid bivalve *Austrovenus stutchburyi*, from sandflats inside the mouth of each estuary. *A. stutchburyi* are the dominant suspension-feeding bivalve found in soft-sediment habitats in harbors and estuaries of New Zealand and are an important commercial and recreational fishery. Five adult *Austrovenus* individuals were collected inside the mouth from each estuary and stored frozen until stable isotope analysis (see below).

### Stable isotope analyses

The bivalve individuals retained for isotope analysis were thawed and foot muscle tissues of individual animals dissected. Individual tissue samples were dried for a minimum of 72 hours at 60°C, ground into a fine powder using a dental amalgamator, and a homogenized aliquot (∼1±0.1 mg) packed into tin capsules under clean laboratory conditions. The grinding capsules were washed with 90% ethanol and rinsed in distilled water three times between every sample to minimize contamination.

The samples were analyzed for ^13^C and ^15^N isotopes and elemental composition (%C, %N) using a PDZ Europa ANCA-GSL elemental analyzer interfaced to a PDZ Europa 20–20 isotope ratio mass spectrometer, IRMS (Sercon Ltd., Cheshire, UK). During analysis, samples are interspersed with replicates of laboratory standards, which have been previously calibrated against Standard Reference Materials. The long-term standard deviation is 0.2‰ for δ^13^C and 0.3‰ for δ^15^N. The final delta values are expressed relative to international standards V-PDB (Vienna PeeDee Belemnite) and Air (N_2_) for carbon and nitrogen, respectively, using the formula:
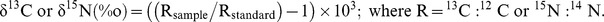


### Data analysis

To estimate the contribution of estuarine-derived production to bivalves inside the estuary and along an estuary-to-coast gradient, we used mass balance isotope mixing models [20] for each site. Five potential primary producers (with values specific for each estuary) were used in the model (estuary SPOM, marine SPOM, seagrass detritus, microphytobenthos, and fringing vegetation detritus). Fringing vegetation was the mean value of saltmarsh and mangrove detritus as these primary producers were functionally similar and did not differ significantly in isotope composition. The mean isotope values of δ^13^C and δ^15^N and the standard error of each potential source, averaged across the estuarine gradient, were used in the models and isotopic ratios were corrected for isotopic fractionation associated with one trophic level using the mean values (δ^15^N = 2.3ä; δ^13^C = 0.4ä) from [21]. The seagrass detritus collected at the offshore site at Whangapoua exhibited highly enriched ^15^N values (δ^15^N = 21.3ä) indicative of bacterial enrichment during degradation. Therefore to account for isotope enrichment during degradation, we used the mean value of fresh seagrass and this seagrass detrital sample (analyzed in triplicate) in the mixing models for Whangapoua estuary (δ^15^N = 15.8ä; δ^13^C = −12.1ä). This averaging takes into account changes in stable isotope discrimination as the food quality changes during degradation [22]. At Tairua, fresh detritus collected within Tairua estuary was used in the mixing model with a correction for the fractionation associated with degradation. We tested possible contributions of the different food sources to *D. subrosea* at each site for which data were available along the estuary-to-coast gradient for both Whangapoua and Tairua estuaries using estuarine specific isotope values, a tolerance setting of 0.1–0.3 and an increment of 1%. Indeterminable solutions are indicated where the tolerance has to be raised above 0.5, the range of feasible solutions are broad ranging from 0% upwards, and there is a high proportion of overlap between source proportions [20]. Model runs producing narrow ranges of feasible solutions indicate a more complete characterization of diet.

A range of linear and non-linear regression models or categorical generalized linear models were used to describe spatial patterns in relation to distance from the estuary mouth as appropriate [23]. Significant differences in stable isotope values of bivalves among sites were determined using one-way ANOVAs and Tukey HSD tests. For the categorical models where a significant difference was detected (P<0.05), Bonferroni multiple comparisons test was used to identify the significance of differences between individual sites.

The species richness estimate, Chao 2 [24], [25] was used to assess macrobenthic taxa accumulation in the core samples. Canonical ordinations were run on both locations, combined and separately, to determine which environmental variables best explained the variability in community composition. Two different techniques were used: canonical correspondence analysis (CCA in CANOCO, [26], which uses a chi-square distance measure and for which forward selection is the only selection process available; and DISTLM (Primer E Permanova+, [27], using Bray-Curtis dissimilarities and backwards selection based on adjusted R^2^ and AIC). For both techniques, raw macrofaunal data and normalized environmental data were used. As both techniques gave very similar results only the DISTLM results are presented here. In building these models we used site location as a rank order surrogate of distance from the mouth of the estuary, the sediment environmental characteristics derived from the core sampling, and information on the size and density of the large *Dosinia* bivalves derived from the quadrat samples.

Functional diversity was characterized by converting the macrobenthic community data into a biological trait matrix containing 32 traits [28], which allowed us to define species traits based on an organism's living position, sediment topographic features created, the direction of sediment particle movement, the degree of motility, feeding behavior, body size, shape and hardness. The abundances of specific traits were summed over taxa at a site and then analyzed using the ordination techniques described above.

## Results

**Figure 1:**
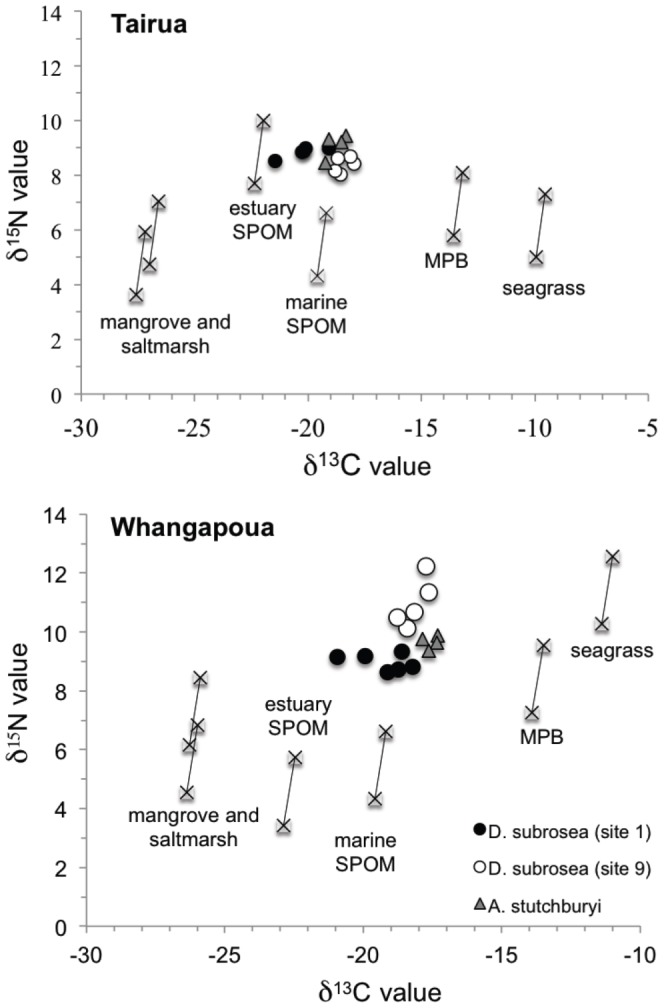
Dual isotope (δ^13^C, δ^15^N) plots of estuarine primary producers (crosses) and predicted fractionation associated with one trophic level for Tairua and Whangapoua estuaries. *Dosinia subrosea* (foot muscle) from site 1 closest to the estuary mouth are presented as solid circles and from site 9 as empty circles. For comparative purposes, the isotope values of a suspension-feeding bivalve, *Austrovenus stutchburyi*, within each estuary are presented as grey triangles.

The primary producers collected within the estuaries and offshore exhibited differences in mean carbon and nitrogen isotope values (Figure 1). Estuarine suspended particulate organic matter (SPOM) was depleted in ^13^C (Whangapoua δ^13^C = −22.9‰, Tairua δ^13^C = −22.4‰) relative to marine SPOM (δ^13^C = −19.6‰), enabling us to partition the uptake of estuarine versus marine SPOM by bivalves. While estuarine SPOM was significantly higher in δ^15^N at Tairua (7.7‰) than marine SPOM (4.3‰) (t_(9)_ = −4.59, p = 0.0037), no significant difference was detected (p>0.05) in mean δ^15^N values for SPOM at Whangapoua (3.4‰) and offshore (∼4‰). Saltmarsh and mangrove leaves were depleted in δ^13^C (<−26‰), while seagrass detritus was relatively enriched in ^13^C (−9‰ to −11‰). Fresh seagrass was significantly enriched in δ^15^N (F_(1, 17)_ = 5.47, p = 0.032) at Whangapoua (>10‰) relative to Tairua (∼7‰). Estuarine microphytobenthos was relatively enriched (δ^13^C≥13‰, δ^15^N≥5.8‰) compared to marine SPOM. *Dosinia subrosea* samples collected offshore near the estuary mouth had similar isotope signatures (p>0.05) to bivalves (*Austrovenus stutchburyi*) collected within each estuary (Figure 1). Further, *D. subrosea* samples collected near the mouth (site 1–8) were significantly depleted in ^15^N relative to samples collected at the furthest sites (site 9) at Whangapoua (F_(8, 37)_ = 7.63, p<0.0001), but these differences in δ^15^N among coastal sites was not evident at Tairua (F_(8, 27)_ = 1.29, p = 0.29). There was a significant difference in bivalve δ^13^C values among sites at Whangapoua (F_(8, 37)_ = 4.35, p = 0.0009), although this was not clearly related to distance from the mouth. At Tairua, there was a trend of increasing δ^13^C values with distance from the mouth, however this was not statistically significant (F_(8, 27)_ = 1.25, p = 0.31).

**Figure 2:**
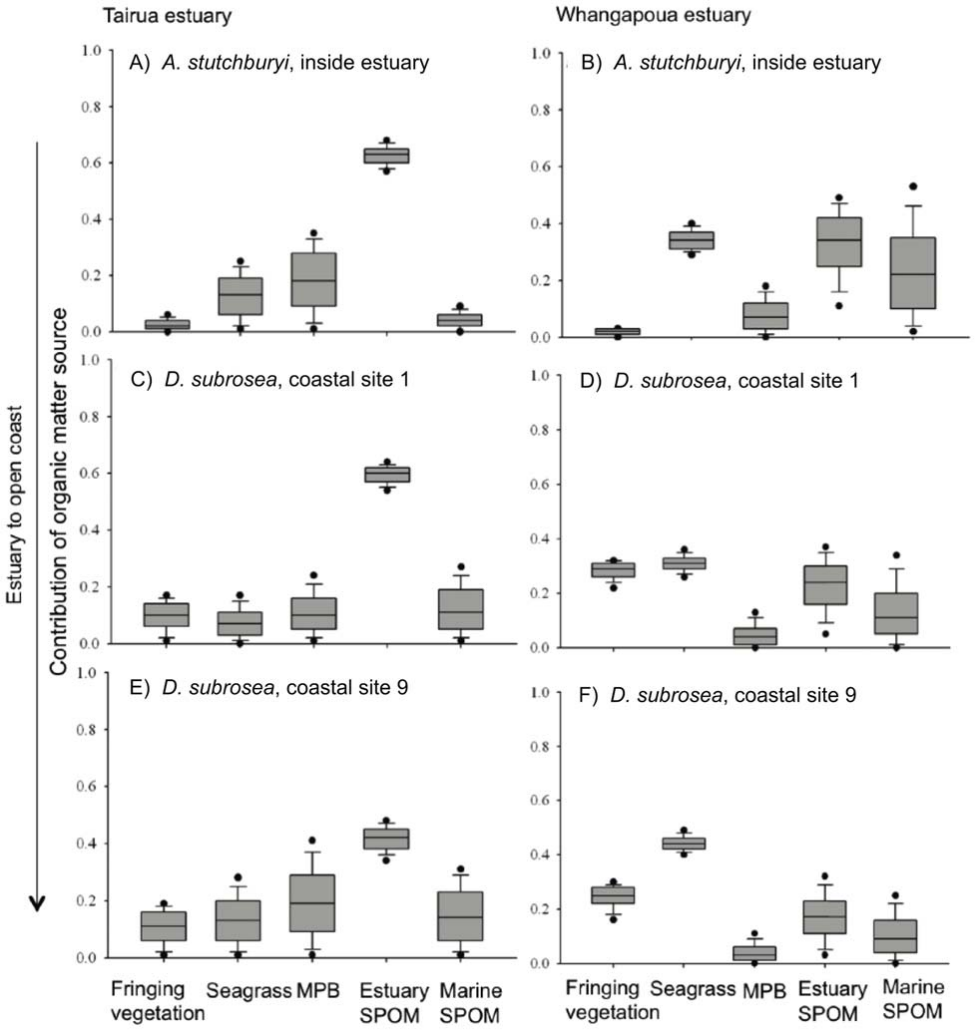
Mixing model outputs showing the range of the potential contribution (0.0 to 1.0 representing 0% to 100%) of different primary producers to venerid bivalves, *Austrovenus stutchburyi* inside the estuaries and *Dosinia subrosea*, on the open coast at Tairua (left) and Whangapoua (right) estuaries. The shaded boxes represent the 75^th^ (top) to 25^th^ (bottom) percentiles with the central lines indicating the median. The vertical lines outside the boxes indicate the 95^th^ (top) and 5^th^ (bottom) percentiles. MPB = microphytobenthos.

Isosource models to estimate resource subsidies demonstrated that estuarine SPOM was the dominant food resource (∼60% near Tairua estuary mouth) that contributed to secondary production of mollusks inside Tairua estuary and on the adjacent coast, and was also an important food source at Whangapoua estuary (Figure 2). Seagrass detritus and, to a lesser extent, fringing vegetation were also important estuarine organic matter sources to bivalves at Whangapoua estuary. Consistent with our hypothesis, there was a decreasing contribution of estuarine primary production to bivalve biomass with distance from the estuary mouth. Estuary SPOM contributed between 58–65% to bivalve (*A. stutchburyi*, *D. subrosea*) biomass near the mouth and between 38–45% (*D. subrosea*) up to 4 km (site 9) from the mouth of Tairua estuary. A similar pattern of decreasing contribution of estuary SPOM to bivalves with distance from the estuary was recorded for Whangapoua estuary.

The two coastal study areas (Tairua, Whangapoua) had low organic content (<2.5%), with only Tairua exhibiting a trend of decreasing concentration moving away from the mouth of the estuary. Both locations were dominated by fine sands, which accounted for >75% of the sediment, and there was a trend of increasing coarse sediment with distance from the estuary mouth at Tairua. Sediment mud content was low across all sites. The sediment comprised 3–5% fine carbonate material at both locations with no clear spatial gradients. An exception was noted at the site furthest from the estuary mouth at Whangapoua, which had anomalously high sediment carbonate content. MPB standing stock in the surface sediments of coastal sites was higher at Tairua (mean chl *a* 9.37 µg g^−1^) than Whangapoua (5.73 µg g^−1^). Further, benthic standing stock was higher (12–13 µg g^−1^) near the mouth of the estuary (sites 3–5) at Tairua. The lower values and slightly higher chlorophyll to phaeophytin ratios indicate a slightly less productive system at Whangapoua than Tairua.

Species richness of the macrobenthic community (derived from the core samples) in the coastal soft-sediments at both locations was high. Macrofauna taxa accumulation curve based on Chao2 ± S.E. demonstrated that diversity appeared to level off after about 20 cores at Whangapoua, while taxa richness continued to increase at Tairua (Fig. S2). This analysis indicated about 90 macrofauna taxa at Whangapoua and in excess of 130 taxa at Tairua. Macrobenthic community composition was different between Tairua and Whangapoua, with a Bray-Curtis dissimilarity of 60%. Within-location similarity was higher at Tairua (74% similarity) than Whangapoua (66% similarity). The numerically dominant taxa at both locations were small polychaetes and crustaceans, which were either highly mobile or build tubes in the sediment and were predominantly surface deposit feeding taxa.

**Figure 3:**
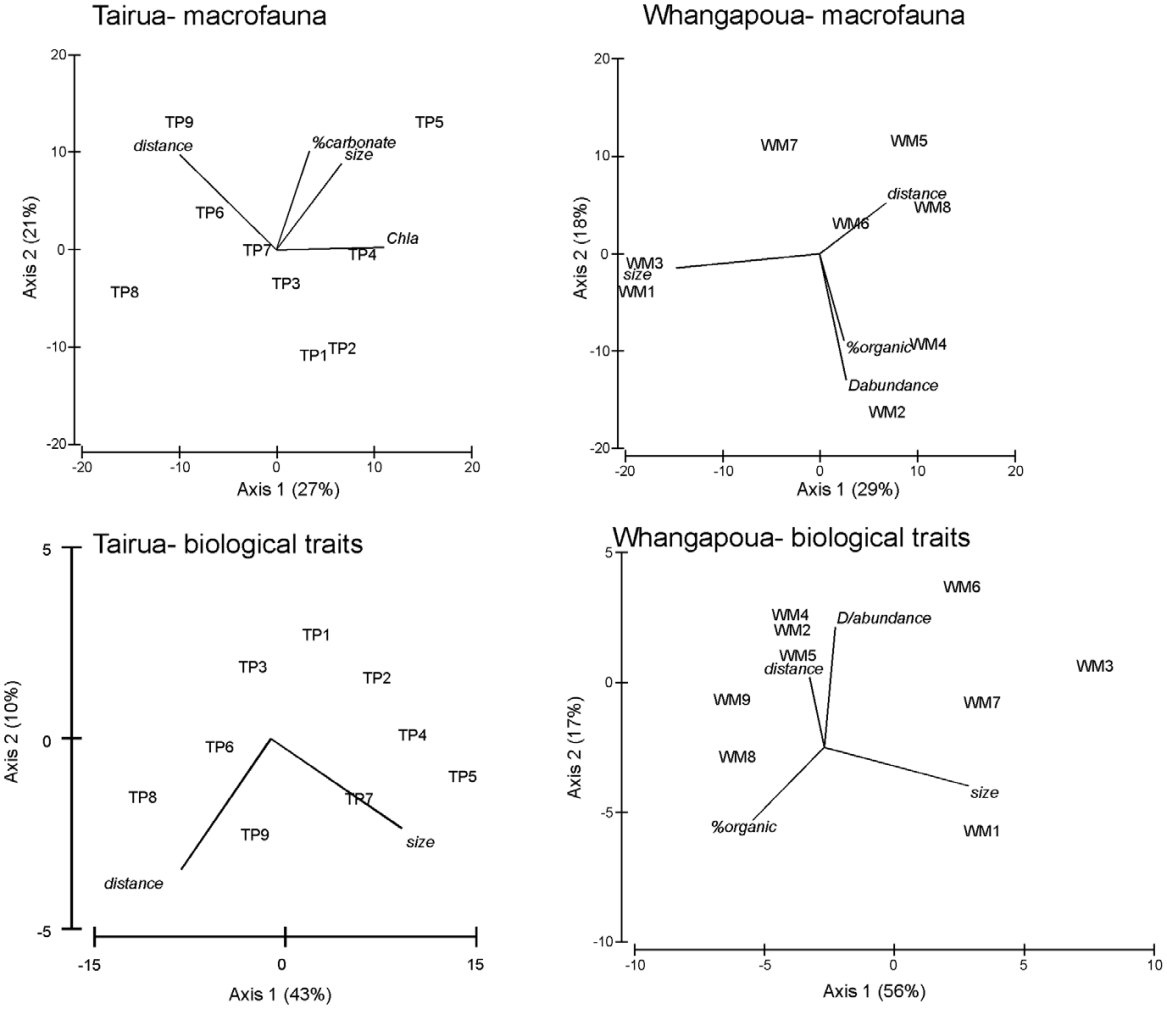
DISTLM ordination results showing the relationship between sites sampled across a gradient from the estuary mouth to ∼4 km offshore and forcing functions for macrobenthic communities (top panel) and biological traits (bottom panel). D/abundance = number of individuals of *D. subrosea*; size refers to the mean size of *D. subrosea* at that site; distance refers to distance from the estuary mouth. Tairua coastal sites are designated as TP1-9 and Whangapoua coastal sites as WM1-9 (Site 1 is closest to the estuary mouth).

At Whangapoua, four variables were sufficient to build the best model (R^2^ = 0.72, distance, mean *Dosinia* abundance, % organic matter and mean *Dosinia* size), relating environmental factors to macrobenthic community composition (Figure 3). Distance from the mouth of the estuary alone explained 16% of the variability in the macrobenthic community composition. At Tairua, four variables were sufficient to build the best model (R^2^ = 0.66, distance, chlorophyll *a*, % sediment carbonate content and mean *Dosinia* size). Distance alone explained 25% of the variability. When both locations were used in the same model, differences between sites, notably in sediment grain size, drive the model.

Distance from the estuary mouth and the size and density of large bivalves (*Dosinia* spp.) were significant drivers of the macrobenthic communities and biological traits at both locations (Figure 3). At Whangapoua, traits that differed along the gradient, with higher abundances further away from the mouth, were large, round-shaped, grazers and suspension feeders and calcified organisms; these traits are commonly expressed by mollusks. At Tairua, there was a reverse effect, with more round-shaped organisms found near the mouth. Higher abundances of freely motile organisms living in the top 2 cm and disturbing the surface were also found near the mouth at Tairua; these traits are commonly exhibited by small crustaceans. Both locations tended to have more deposit feeders and higher abundances of small organisms near the mouth.

*Dosinia subrosea* comprised ∼80% of all large bivalves sampled (derived from the quadrat sampling). Densities of *D. subrosea* were about an order of magnitude higher at Whangapoua than Tairua (Fig. S3), with densities at sites near the Whangapoua estuary mouth particularly high (>35 ind 0.25 m^−2^; significantly higher than the more distant Whangapoua sites). Although the densities of *D. subrosea* were lower at Tairua, the sizes of the *D. subrosea* present were similar to those at Whangapoua. There were no clear trends in *D. subrosea* size or abundance in relation to distance from the estuary mouth at Tairua. The presence of the congener *Dosinia anus* at Tairua, but not at Whangapoua, was consistent with the greater exposure of the Tairua site to ocean swell, as *D. anus* is considered to be the more wave-tolerant species.

## Discussion

Our study demonstrated that estuarine primary production can provide a substantial subsidy to large suspension feeding bivalves in adjacent coastal habitats. The presence of large bivalves and the distance from the estuary mouth both play important roles in determining community composition and the functional diversity of the coastal macrobenthic community. Thus, the resource subsidies that are exported from the estuaries play a role in enhancing biodiversity and ecosystem functioning of coastal systems.

### Estuarine resource subsidies to coastal ecosystems

Estuarine primary production provided food subsidies, contributing to secondary production of the adjacent coastal benthic communities. The stable isotope ratios indicated an important subsidy (up to ∼60% near Tairua estuary mouth) from estuarine primary food resources to large suspension-feeding bivalves offshore (Figure 2). These estuarine resources were important food subsidies to *Dosinia subrosea* over annual timescales (based on ancillary tissue turnover rate data; authors, unpublished), particularly near the estuary mouth. Further, the isotopic signatures of the venerid bivalves (*Dosinia subrosea*) offshore were similar to suspension feeding venerid bivalves (*Austrovenus stutchburyi*) within the respective estuaries (Figure 1), suggesting that similar organic matter sources are utilized and assimilated by both species. Accordingly, our findings support the ‘outwelling hypothesis’ proposed by Odum and colleagues [5], demonstrating that excess estuarine primary production is advected to the adjacent coastal system where benthic communities can take advantage of the higher production.

Fluxes between estuaries and the coastal ocean can form plumes of elevated chlorophyll concentration, bacterial abundance and activity, and nitrification [29], [30], [31]. These estuarine exports of bacterioplankton can in turn stimulate zooplankton, with up to 47% of the carbon demands of zooplankton met by terrestrial and estuarine carbon during pulsed freshwater flows [32]. Thus, while the importance of estuarine organic exports for pelagic coastal food webs has been studied, no published studies have assessed the role of estuarine exports for benthic coastal food webs. Our findings based on bivalve foot muscle tissues suggest that estuarine production provides a substantial food subsidy (up to ∼60%) to sedentary benthos over annual timescales (authors, unpublished data). Thus, the habitat where ecosystem services like productivity are generated are not necessarily where the services are utilized.

There was a gradient of decreasing contribution of estuarine primary production to coastal mollusks with distance from the estuary mouth, consistent with our hypothesis. The estuary-to-coast gradient was strongest for estuarine SPOM where estuarine production contributed ∼60% to the bivalve biomass near Tairua estuary mouth and percent contribution decreased to ∼40% at the furthest site (Figure 2). Similarly, at Whangapoua, estuarine SPOM contributed ∼30% near the estuary mouth but only ∼10% at the furthest site. Tairua has a greater mean annual river discharge (21.2 m^3^ s^−1^) than Whangapoua (6.1 m^3^ s^−1^) but a smaller total volume on spring tide (Tairua: 7.8×10^6^ m^3^; Whangapoua: 17.2×10^6^ m^3^) [16], [17]. Thus, simple geomorphological characteristics of the two estuaries do not account for the differences between Tairua and Whangapoua. At Tairua, there was no significant difference detected (p>0.05) in the isotope values of *Dosinia subrosea* near the estuary mouth and at the furthest site, most likely due to Tairua offshore sites being exposed to a more dynamic oceanic wave environment, whereas the Whangapoua sites were more sheltered (authors personal observation). Thus, we propose that the greater wave energy off Tairua blurs the estuarine organic matter signature offshore. By contrast, bivalves analyzed off Whangapoua estuary mouth (sites 1–8) were distinct from those sampled at site 9 furthest from the estuary mouth (Figure 1). Nonetheless, resource subsidies from the estuary were still detected up to 4 km from the mouth of the estuary at both locations, highlighting the importance of estuarine subsidies to the open coast.

The type of estuarine food resource supporting secondary production on the adjacent coast varies between locations that were only ∼50 km apart and this likely has an effect on the structure and function of the benthic communities. At Tairua, estuarine SPOM was the dominant organic matter source that supported secondary production offshore, while at Whangapoua, there was a greater export of seagrass and fringing vegetation detritus that was both visually observed in the case of seagrass and supported by the stable isotope values of *D. subrosea* (Figure 2). Tairua coastal sites, which received a significant contribution of estuarine SPOM (e.g., up to 65% near the mouth) compared to Whangapoua (about 10–30%), had higher species richness than Whangapoua macrofaunal communities (data not presented). Estuarine-derived SPOM likely contained a mixture of estuarine plankton and resuspended microphytes that was advected to the open coast and provided a high quality food resource [33] to the suspension feeding surf clams (*Dosinia* spp.) and the smaller benthic organisms that tended to feed at the sediment-water interface. By contrast, Whangapoua, which is a larger estuary and contains substantial seagrass beds (23% of the surface area of the harbor), exported larger quantities of seagrass detritus to the offshore benthic communities. Seagrass is more refractory than plankton and microphytobenthos and must be degraded by bacteria before it becomes available to suspension-feeding bivalves [34]. This was also supported by our isotope values, which showed substantial enrichment in ^15^N of the detrital seagrass offshore. Therefore, while further research is needed to determine the quality as well as the quantity of the estuarine food subsidy, our results suggest that both factors may be important drivers of species richness and functional trait diversity along estuary-coast gradients.

### Estuaries and large bivalves affect macrofaunal trait composition

Macrobenthic community composition and the functional traits of resident species were strongly influenced by distance from the estuary and the size and density of large suspension feeding bivalves (Figure 3). Distance from the mouth of the estuary alone explained 25% and 16% of the variance in the macrobenthic communities at Tairua and Whangapoua, respectively. Densities of large individuals of the surf clam *Dosinia subrosea* were an order of magnitude greater at Whangapoua than Tairua, particularly near the estuary mouth, however, there was no significant difference in mean size of the bivalves between locations. Macrofaunal species richness was high in both locations, with highest richness recorded at Tairua. The numerically dominant macrobenthic taxa were surface deposit feeding organisms adapted to mobile sediments and patchily distributed food resources, consistent with the hydrodynamic environment of these coastal habitats.

In addition to distance from the estuary mouth and the presence of large bivalves, the structure and function of the benthic communities were influenced by the sediment characteristics and the quantity and quality of organic matter available. Tairua coastal sites had higher *in situ* production, with a trend of slightly higher sediment chl *a* concentrations closer to the estuary with an average of 11.5 µg g^−1^ at sites 1–5, close to the estuary mouth and 6.7 µg g^−1^ at sites 6–9. Further, stable isotope ratios suggest that coastal communities off Tairua had a higher quality food resource exported from the estuary than Whangapoua.

### Conclusions

Estuaries provide important resource subsidies and contribute to secondary production of mollusk-dominated communities in open coast ecosystems. The spatial extent of influence of the two small estuaries we studied extend at least to 4 km from the mouth of the estuary. The trophic connections have effects on coastal biodiversity and the array of functional traits of the macrofaunal community. Maintaining this biodiversity requires integrative ecosystem-based management that is focused on maintaining the resilience of coastal ecosystems.

## Supporting Information

Figure S1 Subtidal study sites off Tairua (left) and Whangapoua (right) estuaries. Site 1 is located on the margin of the estuary discharge channel (depicted with thin black lines). Site 9 is located up to 4 km from the estuary mouth.
(TIFF)

Figure S2 Macrofauna taxa accumulation curve based on Chao2 ± S.E. for Tairua (black) and Whangapoua (grey). At each location, macrofauna were identified and enumerated from the 15 sediment cores taken at each of the 9 study sites.
(EPS)

Figure S3 Mean densities of large *Dosinia subrosea* (+SE) derived from quadrat samples in relation to distance from estuary mouth at Tairua (open blocks) and Whangapoua (shaded blocks). Site 1 is closest to each estuary mouth. The significance of differences between sites in each location is based on a one-way GzLM with site as a fixed factor. A corrected Bonferroni multiple comparisons test showed that sites 1–4 at Whangapoua had higher abundances than Whangapoua sites 7–9.
(TIFF)
