# Supplementary figures and images for: Ecosystem Services Transcend Boundaries: Estuaries Provide Resource Subsidies and Influence Functional Diversity in Coastal Benthic Communities
Source: PLoS One. 2012 Aug 3;7(8):e42708. doi: 10.1371/journal.pone.0042708 (PMC3411827; doi:10.1371/journal.pone.0042708)

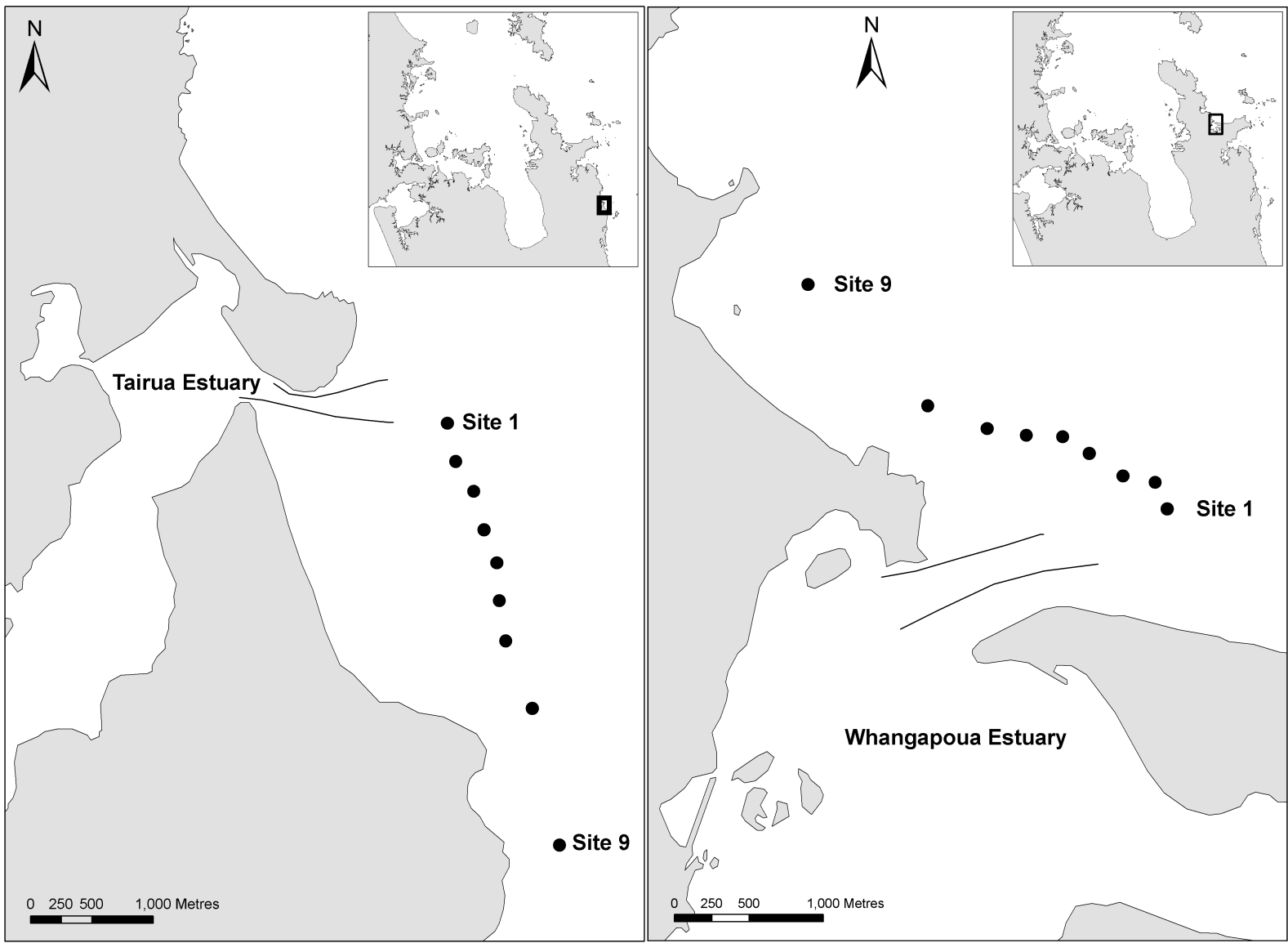

Supplement: Figure S1 — Subtidal study sites off Tairua (left) and Whangapoua (right) estuaries. Site 1 is located on the margin of the estuary discharge channel (depicted with thin black lines). Site 9 is located up to 4 km from the estuary mouth. (TIFF) [file pone.0042708.s001.tiff]

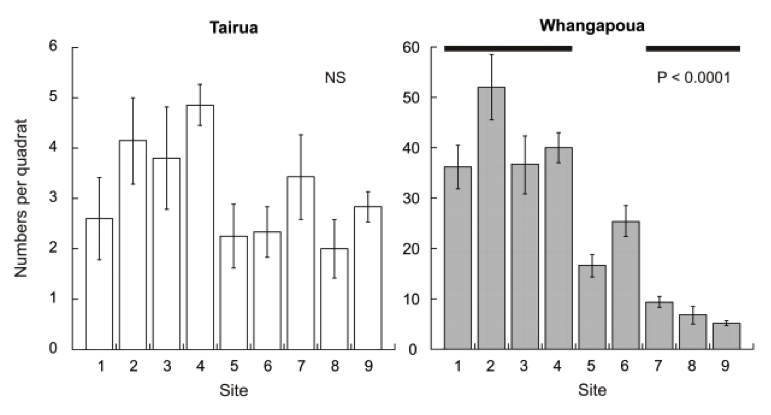

Supplement: Figure S3 — Mean densities of large Dosinia subrosea (+SE) derived from quadrat samples in relation to distance from estuary mouth at Tairua (open blocks) and Whangapoua (shaded blocks). Site 1 is closest to each estuary mouth. The significance of differences between sites in each location is based on a one-way GzLM with site as a fixed factor. A corrected Bonferroni multiple comparisons test showed that sites 1–4 at Whangapoua had higher abundances than Whangapoua sites 7–9. (TIFF) [file pone.0042708.s003.tiff]
